# Reduction of female copulatory damage by resilin represents evidence for tolerance in sexual conflict

**DOI:** 10.1098/rsif.2014.1107

**Published:** 2015-03-06

**Authors:** Jan Michels, Stanislav N. Gorb, Klaus Reinhardt

**Affiliations:** 1 Department of Functional Morphology and Biomechanics, Institute of Zoology, Christian-Albrechts-Universität zu Kiel, Am Botanischen Garten 1–9, 24118 Kiel, Germany; 2 Biological Oceanography, GEOMAR Helmholtz Centre for Ocean Research Kiel, Düsternbrooker Weg 20, 24105 Kiel, Germany; 3 Applied Zoology, Department of Biology, Technische Universität Dresden, Helmholtzstraße 10, 01217 Dresden, Germany; 4 Department of Animal and Plant Sciences, University of Sheffield, Western Bank, Sheffield S10 2TN, UK

**Keywords:** antagonistic coevolution, bed bug, resilin, sexual conflict, tolerance, traumatic insemination

## Abstract

Intergenomic evolutionary conflicts increase biological diversity. In sexual conflict, female defence against males is generally assumed to be resistance, which, however, often leads to trait exaggeration but not diversification. Here, we address whether tolerance, a female defence mechanism known from interspecific conflicts, exists in sexual conflict. We examined the traumatic insemination of female bed bugs via cuticle penetration by males, a textbook example of sexual conflict. Confocal laser scanning microscopy revealed large proportions of the soft and elastic protein resilin in the cuticle of the spermalege, the female defence organ. Reduced tissue damage and haemolymph loss were identified as adaptive female benefits from resilin. These did not arise from resistance because microindentation showed that the penetration force necessary to breach the cuticle was significantly lower at the resilin-rich spermalege than at other cuticle sites. Furthermore, a male survival analysis indicated that the spermalege did not impose antagonistic selection on males. Our findings suggest that the specific spermalege material composition evolved to tolerate the traumatic cuticle penetration. They demonstrate the importance of tolerance in sexual conflict and genitalia evolution, extend fundamental coevolution and speciation models and contribute to explaining the evolution of complexity. We propose that tolerance can drive trait diversity.

## 1. Introduction

**Figure 1.**
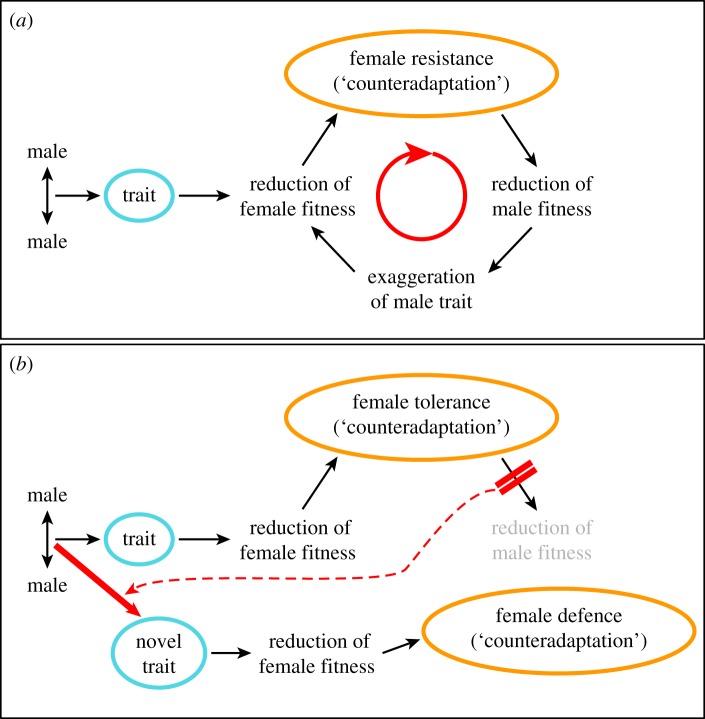
Sexual conflict, trait exaggeration and trait diversification. (*a*) The basic Rice–Holland model [6] of evolutionary conflicts, exemplified by the arms race of a male persistence trait having arisen from male–male competition, selects for a female resistance trait. The perpetual coevolutionary cycle (red arrow) between male harm and harm amelioration leads to trait exaggeration. (*b*) An extended Rice–Holland model, now incorporating female tolerance as a female defence trait. Female tolerance produces no selective feedback on males (double red bars) and can, therefore, facilitate the evolution of a novel male trait (large red arrow). As the original male trait is not selected against, it persists in the population and so increases complexity of the reproductive system. Compared with the basic model, there are now twice as many male offence (blue) and female defence traits (orange).

Sexual conflict arises when the evolutionary interests of males and females diverge and both sexes maximize their fitness by different trait optima [1,2]. Particularly impressive, and widespread, examples of sexual conflict include those male reproductive traits that cause injury to females during mating [3–5]. If females cannot avoid superfluous mating, they are predicted to evolve resistance traits that reduce the damage incurred by mating [1–4,6,7]. The existence of resistance traits is fundamental to theoretical models showing that damaging male persistence and female resistance traits coevolve in arms races and thereby lead to (i) rapid trait exaggeration [1–3,6–12] (Rice–Holland model, figure 1*a*), (ii) novel male and female traits [9], and (iii) rapid emergence of biological diversity [8,13–16].

The current theory features two main problems. First, experimental manipulations showing that discrete behavioural, morphological or physiological female traits represent specific adaptations to reducing the damage imposed by males are rare and difficult (but see [12,17–19]). Among other reasons, this rarity stems from the fact that the very function of female defence, the reduction of harm, evolves rapidly to ameliorate the damage imposed by the male. Consequently, current costs to females are small, unless the ancestral situation without female defence is resurrected experimentally [2,3,6,7,12]. Second, sexual conflict research has only considered resistance as a defence strategy, not tolerance [4,16] (but see [2,10] for similar concepts). Tolerance, as defined for interspecific evolutionary conflicts, is the balance or ‘repair’ of damage without harming the offending party [20–23]. In host–parasite and plant–herbivore conflicts defence by tolerance produces completely different evolutionary dynamics than defence by resistance [20–23]. For example, under tolerance, the lack of harm to the offending party can even result in a lack of coevolution [2,10]. The distinction between tolerance and resistance is important because resistance is known to result in the exaggeration of a trait but it is not clear if resistance can also lead to the evolution of novel traits and thereby to diversification.

**Figure 2.**
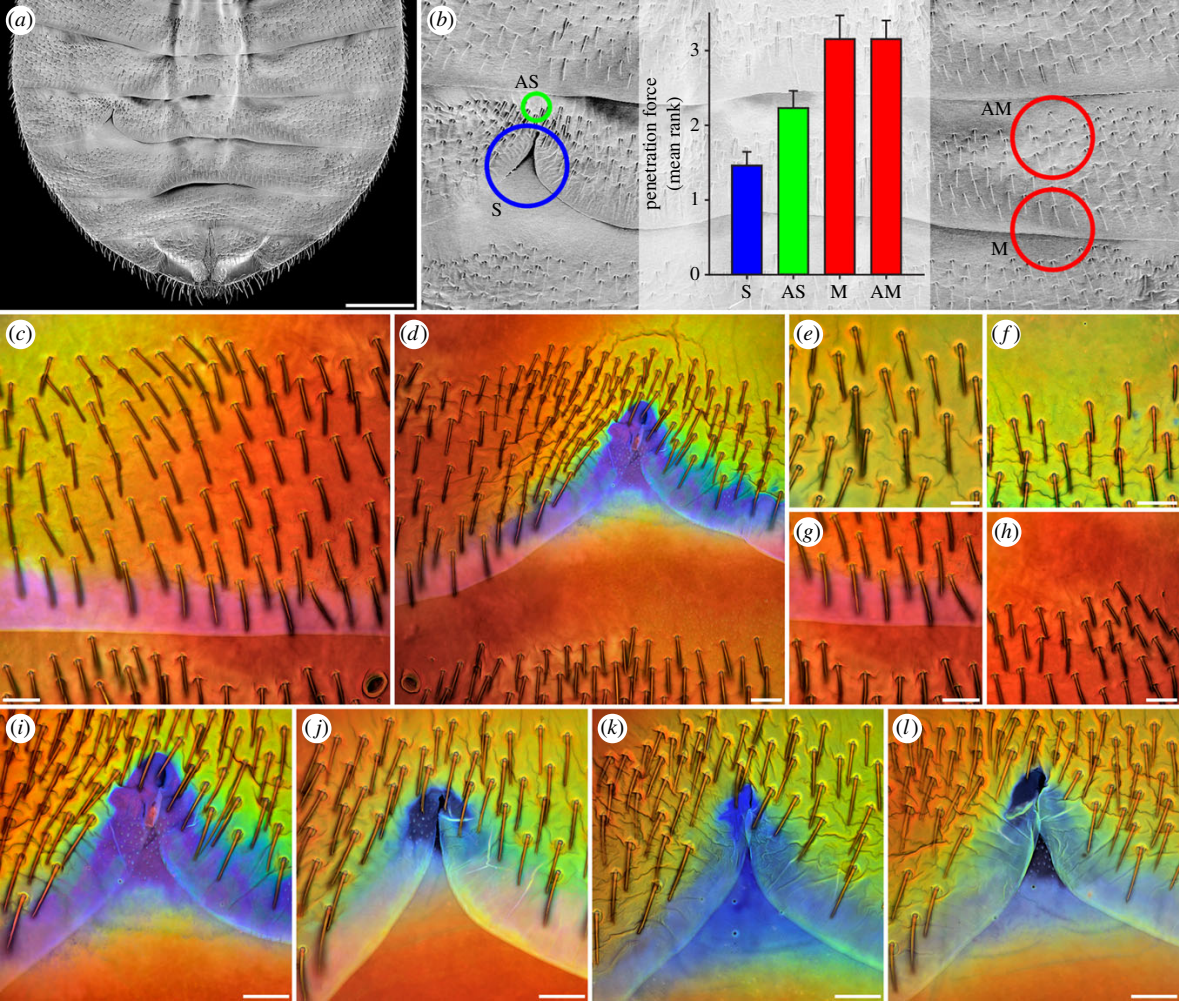
Material composition and properties of the ventral abdominal cuticle of *C. lectularius* females. (*a*) Abdomen overview (scanning electron micrograph). (*b*) Section of (*a*) indicating the locations of the spermalege (S), AS, M and AM, and penetration forces (mean ranks and standard errors, see Material and methods) determined for these four cuticle sites. (*c*–*l*) CLSM maximum intensity projections. (*c*,*d*) Autofluorescence composition of the cuticle in the left (*c*) and right (*d*) abdomen parts. The dominance of violet/blue autofluorescence (shown in blue) is restricted to the spermalege, clearly indicating that only at this site the cuticle contains large proportions of resilin. (*e*–*h*) Autofluorescence composition of the cuticle at the sites AS (*e*,*f*), M (*g*) and AM (*h*). The cuticle at M and AM consists mainly of sclerotized chitinous material, indicated by the dominance of red autofluorescence, while the presence of large proportions of green autofluorescence in the cuticle at AS indicates that the respective material consists mainly of weakly or non-sclerotized chitinous material. (*i–l*) Autofluorescence composition of the cuticle at the spermaleges of different one-week-old females, indicating variation of the extent of the resilin-dominated spermalege structures between females. Scale bars, (*a*) 500 µm, (*c*,*d*,*f*–*l*) 50 µm, (*e*) 25 µm.

To test the possibility that tolerance exists in intraspecific conflicts, we used a textbook example system of sexual conflict, the so-called traumatic mating. This phylogenetically widespread behaviour [3–5] has in bed bugs allowed for the experimental circumvention of female defence [17–19]. Briefly, during every mating, the male penetrates the cuticle on the ventral side of the female's abdomen with a cannula-like intromittent organ, causing a wound, and injects sperm and accessory gland fluids directly in the body cavity [24]. This type of mating carries survival costs to the females [25] but the females are unable to avoid mating [26]. As a result, a novel female organ, called the spermalege, has evolved in the bed bug family Cimicidae. In common bed bugs (*Cimex lectularius*), the spermalege is located on the right ventral side of the abdomen where the males usually penetrate the cuticle. Externally, this organ is apparent as a notch-like modification of the posterior edge of the fifth segment exposing the intersegmental membrane and the cuticle of the sixth segment underneath (figure 2*a*,*b*; see also e.g. [25]). Experimentally circumventing the spermalege and thereby mimicking the ancestral state of the female (i.e. to experience a wound without the counteradaptation) is possible by piercing the female through the left ventral side of the abdomen where no spermalege exists. Such experimental manipulations have indicated that the spermalege has evolved in response to male harm and represents a defence trait that reduces female wounding, water loss and sexual infection [17–19]. This led us to hypothesize that specific properties of the cuticle material inside the spermalege are responsible for the reduction of the male-inflicted damage in the females. We analysed the composition and properties of the spermalege material of *C. lectularius* females and compared them with those of the materials of (i) developmentally equivalent sites in nymphs and males (nymphs and males are not traumatically inseminated in nature, do not possess a spermalege and therefore should not show any defensive material properties) and (ii) control sites within each female. With a resistance function, the properties of the spermalege material should make it more difficult for the males to mate, while, by contrast, with a tolerance function, these material properties should not cause a difference, or make penetration easier. Accordingly, our test represents the first empirical distinction between tolerance and resistance in an intraspecific conflict. The discovery of female tolerance to male harm would require an extension of current coevolution models [6] to include tolerance as a potential mechanism that facilitates the evolution of novel traits, rather than the exaggeration of existing traits.

## 2. Material and methods

### 2.1. Bed bug stock and culture conditions

The bed bugs were taken from a large stock culture at the University of Sheffield. The stock was kept at 26°C and 70% relative humidity and maintained as described previously [18,27].

### 2.2. Microscopic analyses of the ventral abdominal cuticle

Analysing the autofluorescence (i.e. the natural fluorescence) composition has proved to efficiently reveal differences in the material composition of arthropod cuticle structures [28]. We applied this method by using both wide-field fluorescence microscopy (WFM) and confocal laser scanning microscopy (CLSM). The composition and properties of the spermalege material of *C. lectularius* females were analysed and compared with those of the materials of developmentally equivalent sites in nymphs and males and of three other female ventral abdomen sites through which the females are never inseminated and which therefore should lack defensive material properties: AS (anterior to the spermalege, in the fifth segment), M (mirror site opposite to the spermalege) and AM (anterior to the mirror site, in the fifth segment) (figure 2*b*). The bed bug abdomina were separated, embedded and mounted in the same way as described in a previous study [28]. The presence, distribution and intensity of resilin autofluorescence in the abdomina of 15 females, 15 males and 15 nymphs were visualized with WFM as described earlier [28]. CLSM was applied to analyse the material composition of the ventral abdominal cuticle of 25 female bed bugs. Only individually reared, recently eclosed (less than 14 days), clean and fresh females were used for these analyses. We applied the same confocal laser scanning microscope system (Zeiss LSM 700, Carl Zeiss Microscopy GmbH, Jena, Germany) and equipment and determined and used the optimal settings in the same way as described previously [28]. The only difference was that the autofluorescence excited with 639 nm was detected using a longpass emission filter transmitting light with wavelengths longer than or equal to 640 nm instead of a longpass emission filter transmitting light with wavelengths longer than or equal to 560 nm, but this did not have any influence on the results.

Scanning electron microscopy was used to create an overview micrograph showing the ventral side of the abdomen of a female bed bug. In this context, the abdomen was critical-point dried, mounted, sputter-coated and visualized with the same materials and instrumentation and the same settings as described in an earlier study [29].

### 2.3. Microindentation analyses

#### 2.3.1. Resistance to penetration

By forcing a steel pin through the female bed bug abdomen, we determined the force necessary to penetrate the cuticle. Fourteen virgin females were fixed to an object slide with the dorsal side of their abdomen, and the ventral side of their abdomen was penetrated through the four different abdomen sites described above (spermalege, AS, M and AM). The steel pin was a standard insect pin, whose tip mimicked the size and the shape of the distal part of the male intromittent organ. The penetration force was measured in the same way and with the same instrumentation as described earlier [30]. Our experimental design was a repeated measurements design of an individual at four different sites. Using R v. 2.14.2 (The R Foundation for Statistical Computing, http://www.r-project.org) we applied a generalized mixed effect model (glmer) in which the penetration force was modelled as a function of the penetration site (fixed effect) within female identity as random effect [31]. The random effect was small (variance between females = 0.0779, variance within females = 0.5595), i.e. individual females varied overall in their cuticle thickness. Part of the variation between females was explained by the female body size: the body size was positively correlated with the penetration force at the sites M and AM (*r*(M) = 0.839, *p* < 0.001; *r*(AM) = 0.583, *p* < 0.025) but not at the body half where the copulation organ is situated (*r*(S) = 0.158, *p* > 0.5; *r*(AS) = −0.233, *p* < 0.25). For figure 2*b*, we ranked the penetration force per penetration site within females (*n* = 13; in one female no result was obtained for one of the penetrated sites) and compared the mean rank per penetration site to account for between-female differences.

#### 2.3.2. Haemolymph loss

During each of the indentation trials, we recorded whether haemolymph exuded from any of the four indentation sites after the withdrawal of the needle.

#### 2.3.3. Wounding damage

To test if a lower penetration force results in reduced tissue damage, we compared the degree of melanization (a general measure of tissue damage in insects [32]) occurring at the spermalege with that occurring at the site M after repeated wounding. For this, we used ethanol-fixed females from a previous study [18]. Each of these females had undergone an identical mate-mimicking procedure performed with sterile glass needles that had been pierced through the abdomen sites at a rate of four penetrations per week [18]. Wounding was characterized at a categorical scale ranging from 1 (very small localized melanin occurrence = small tissue damage) to 6 (entire abdomen melanized = large tissue damage) with the following categories: 1 = small black marks at the piercing site, 2 = one large black mark around the piercing site, 3 = entire segment of the piercing site black, 4 = two to three segments black, 5 = four segments black, 6 = entire abdomen black. The results obtained for the two abdomen sites were compared using the Welch's *t*-test.

### 2.4. Spectral imaging of the resilin autofluorescences

To get more precise information about the resilin proportion in the spermalege material, we compared the spectral properties of the resilin autofluorescence in the spermalege with those of the resilin autofluorescence exhibited by an elastic dragonfly tendon. The so-called elastic tendons of the pleuro-subalar muscles in dragonflies of the genus *Aeshna* consist almost exclusively of resilin (99%; see [33]) and feature the highest resilin proportion having been found in nature so far. Therefore, they are the ideal reference structures in the context of spectral analyses of resilin autofluorescences. For such analyses, the elastic tendons from the hind wing systems of male *Aeshna cyanea* and the abdomina of female *C. lectularius* were prepared, embedded and mounted as described previously [34]. The emission spectra of the resilin autofluorescences exhibited by these structures were analysed by applying the confocal laser scanning microscope system Zeiss LSM 710 (Carl Zeiss Microscopy GmbH) equipped with the inverted microscope Zeiss Axio Observer and a diode pumped solid-state ultraviolet (UV) laser (60 mW, operated at 20 mW) with a wavelength of 355 nm. For the analyses of the elastic tendons, a 10× objective (Zeiss Plan-Apochromat, numerical aperture (NA) = 0.45) and a laser power of 15% were used, while the spermaleges were analysed with a 20× objective (Zeiss Plan-Apochromat, NA = 0.8) and a laser power of 25%. Optical sections (pinhole size = 1 Airy unit) through the structures of interest were visualized with an image size of 1024 × 1024 pixels and a line average of 2. Detector gain and scan velocity were adjusted in a way resulting in a very good signal-to-noise ratio and maximum intensities of the autofluorescence signals detected at the emission maximum while simultaneously preventing any oversaturation. Digital gain and digital offset were set to 1 and 0, respectively. The emission ranges 403–725 nm (elastic tendons) and 414–725 nm (spermaleges) were analysed in detail by detecting emission signals from 32 (elastic tendons) and 33 (spermaleges) equal sections simultaneously with 32 and 33 channels, respectively, resulting in lambda stacks. The results were processed with the software Zeiss Efficient Navigation (ZEN) 2011 (Carl Zeiss Microscopy GmbH) to display the emission spectra and to create lambda-coded images of the optical sections. Two mixed autofluorescence signals exhibited by each of the elastic tendons when exposed to the 355 nm laser light were segregated by applying the linear unmixing function of ZEN 2011.

### 2.5. Measurement of the male costs of mating

We tested if penetration through the spermalege imposes fitness costs on the male bed bugs. In three replicate trials, virgin males and females were generated by isolating last instar nymphs [18]. In each trial, the males were allocated to two treatments with two levels each, and male costs were measured after imposing a stress protocol on them. The males were mated for 60 s [18,26] to either one or four females within a week. We accounted for the fact that females can, to a certain degree, execute behavioural resistance to mating. Females that are not fed and have a flat body can withstand mating in some cases, whereas females that are fed and have bloated, blood-engorged bodies cannot [26]. The males were, therefore, provided with either fed or non-fed females. After mating, the males were transferred to individual vials and kept under stressful conditions, i.e. at 35 ± 1°C (range) at a relative humidity of 16%. The lifespan was recorded in 2-day intervals. The three replicate trials differed in the age of males at the beginning of the trials. We acknowledge that costs to males involve other costs than penetration and that the presence of such costs would be inclusive. However, the absence of costs would not be consistent with resistance, but with tolerance. Variation in the male lifespan was analysed using Cox regression, i.e. Cox's proportional hazard model implemented in R v. 2.15.1 [35] (coxph) whereby the lifespan was analysed as a function of the mating rate (one versus four matings per week), female behavioural resistance (0 versus 1) and the interaction between them. After fitting a full model with trial as stratum and its interaction with the main effects, we applied model reduction procedures based on AIC and *p-*values [31]. The minimum adequate model contained both main effects and no interaction between them (lifespan ∼ strata(trial) + mating rate + behavioural resistance; *n* = 130, seven censored data points).

## 3. Results and discussion

When exposed to UV light by means of WFM, females, males and nymphs exhibit a violet/blue autofluorescence in the intersegmental membranes. This autofluorescence indicates the presence of the rubber-like elastic protein resilin in the respective structures [36,37]. In females, the resilin autofluorescence is also present in the notch-like structure of the spermalege and in a small cuticle area of the sixth segment posterior to this notch-like structure. By contrast, neither the female sites AS, M and AM nor any of the four sites in males and nymphs exhibit such an autofluorescence.

Our CLSM analyses confirmed the presence of resilin in the intersegmental membranes. However, they additionally revealed that overall the membrane material is not dominated by this protein and that other cuticle components are also present in considerable proportions (figure 2*c*,*d*). By contrast, the material of the entire spermalege, including the intersegmental membrane within the spermalege, contains large proportions of resilin (figure 2*d*,*i*–*l*). The extent of the resilin-dominated structures in the spermalege varies between females of the same age (figure 2*i*–*l*) but all females analysed in this study possessed such structures. At the control sites M and AM, the cuticle material is dominated by sclerotized chitinous material (figure 2*g*,*h*), whereas the cuticle material at the control site AS, located just anterior to the spermalege, consists mainly of weakly or non-sclerotized chitinous material (figure 2*e*,*f*).

**Figure 3.**
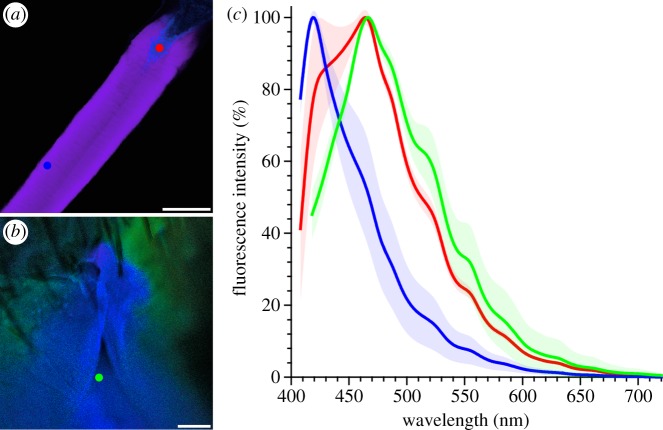
Properties of resilin autofluorescences. (*a*,*b*) Lambda-coded optical sections through a large part of the resilin-dominated structures of an elastic tendon of *A. cyanea* (*a*) and through a spermalege area of *C. lectularius* (*b*), showing autofluorescences excited by 355 nm laser light. In most of the tendon structures, violet resilin autofluorescence is dominant, while the terminal tendon parts exhibit mainly blue resilin autofluorescence. The spermalege is dominated by autofluorescence that is similar to the blue autofluorescence in (*a*), which indicates a resilin proportion similar to that in the terminal tendon parts. The surrounding cuticle exhibits green autofluorescence, indicating large proportions of other cuticle components such as chitin. The dots indicate locations where the fluorescence emission properties were analysed. (*c*) Emission spectra of the resilin autofluorescences exhibited by the elastic tendon (blue and red) and the spermalege (green); colours correspond to the dot colours in (*a*,*b*); mean values (lines, *n* = 5) and standard deviations (shaded areas). The spectral properties of the tendon's violet resilin autofluorescence are similar to those described for the ‘typical’ resilin autofluorescence [36,37], while the properties of the tendon's blue resilin autofluorescence are similar to those of the resilin autofluorescence present in the spermalege. Scale bars, (*a*) 100 µm, (*b*) 25 µm.

The spectral analysis revealed that the resilin autofluorescence present in the spermalege is similar to the resilin autofluorescences exhibited by certain parts of the elastic tendon (figure 3) and by resilin-dominated structures of copepod gnathobases described earlier [34]. This similarity suggests that, compared with the tendon parts featuring the largest resilin proportions, the resilin-dominated structures of the spermalege contain only slightly lower proportions of resilin and slightly higher proportions of chitin, which is typically present in the form of lamellae and fibres within resilin structures [33,36,37] (see also [34]).

The combination of the essential role of the spermalege in the mating process and the fact that no comparable structures with similar material compositions are present in males and nymphs suggested to us that the high resilin proportions in the spermalege might have evolved specifically in response to the traumatic mating. Compared with chitinous cuticle material, resilin is very soft [29,38]. We hypothesized that this softness can (i) reduce the force the male must apply to successfully penetrate the spermalege and thereby (ii) decrease the tissue damage caused by the mating. We tested both predictions by using microindentation. Penetrating the spermalege, on average, required a force of 1.25 ± 0.23 (SE) mN. Confirming our prediction, significantly higher forces were necessary to penetrate the other three cuticle sites (figure 2*b*; AS: 1.98 ± 0.16 (SE) mN; M: 2.74 ± 0.27 (SE) mN; AM: 2.35 ± 0.21 (SE) mN; generalized mixed effects model, adjusted for individual differences between females (see Material and methods): AS: *t* = 2.366, *p* = 0.0178; M: *t* = 4.467, *p* = 0.0004; AM: *t* = 3.200, *p* = 0.0038). In addition, the penetration force measured at AS was lower than those measured at M and AM (figure 2*b*), which is in agreement with our CLSM results indicating the presence of weakly or non-sclerotized and, therefore, very likely softer chitinous cuticle material anterior to the spermalege. To test the second prediction, we compared the degree of melanization (a general measure of tissue damage in insects [32]) at the spermalege with that at the site M in a series of *C. lectularius* females that had been subjected to four weekly experimental penetrations in an independent study [18]. We found a significantly lower mean melanization rank for the spermalege (3.38) than for M (4.40) (95% confidence interval of the difference between the two: 0.180, 1.858; *t*_30.005_ = 2.4804, *p* = 0.019). Although there might be other reasons for differences in the melanization, these results are consistent with the wounding reduction function of the spermalege observed in an earlier study [17].

Resilin is also an extremely resilient material [36,37]. Therefore, we predicted that the high resilin proportions in the spermalege might seal the abdomen puncture after the withdrawal of the male intromittent organ. This hypothesis was motivated by previous results showing that water loss from the body had been reduced when the spermalege had been experimentally punctured, whereas puncturing other abdomen sites had not had any influence [19]. We tested this possible explanation by recording haemolymph leakage from the four abdomen sites after the removal of the indenter. Supporting the sealing hypothesis, no haemolymph leakage occurred at the spermalege and in its vicinity at the site AS, whereas it was frequent at the sites M (27%) and AM (40%).

None of the spermalege defence functions identified by us evidently represents resistance. Instead, the facilitated, rather than an impeded, penetration by the male is in direct opposition to resistance, and all effects are consistent with a tolerance function. We, therefore, tested another key difference between tolerance and resistance directly. If the spermalege evolution has proceeded via resistance, the penetration through the spermalege is predicted to impose fitness costs on mating males. By contrast, no such costs are predicted by the tolerance model. Inconsistent with female resistance, but consistent with female tolerance, we found no evidence across three different-aged male cohorts that a higher penetration (mating) rate (with both behaviourally resistant and behaviourally non-resistant females) increased the survival costs to males (Cox's proportional hazards 0.922 [CI 0.818, 1.040, *p* = 0.189]).

Our study, linking functional morphology and biomechanics with evolutionary biology, strongly indicates that, in the context of the female defence against male cuticle penetration, resilin seals the sexually imposed wounds and physically facilitates copulation by males. This facilitation has two important conceptual consequences. First, the facilitation of penetration results in reduced tissue damage in the females. At a general level, this observation supports theoretical models showing that sexual conflict can generate cooperation [11]. Applied more specifically to the key question in current models of genitalia evolution [39,40], our data suggest that seemingly cooperative female genitalic structures, such as those facilitating penetration, cannot automatically be regarded as products of cryptic female choice [41].

The second important implication of the resilin-mediated physical facilitation of cuticle penetration relates to current models of sexual conflict. The lack of measurably increased survival costs to males with higher mating frequencies supports the idea that female defence against male harm can evolve in the form of tolerance, and not necessarily always in the form of resistance. Given the absence of costs of female defence to males, we propose that sexual conflict models will benefit from considering female tolerance to male harm. While persistence–resistance cycles select for trait exaggeration [6], a lack of negative feedback on males can have the dramatic consequence that coevolution is halted and consequently not necessarily leads to the predicted trait exaggeration [6] or reproductive isolation [8]. However, our results can be used to provide an extended Rice–Holland model of sexual coevolution (figure 1*b*). Female tolerance prevents male trait exaggeration (figure 1*b*, double red bars). As selection still scans the male genome constantly for adaptations that increase male reproductive success [9], the lack of female resistance can facilitate selection for novel male traits in a population (figure 1*b*, large red arrow). Under this extended Rice–Holland model, variation in males increases. Because the male trait is not selected against and persists in the male, the extended Rice–Holland model can explain the evolution of complexity, in particularly the intertwined manifold interactions between males and females typically observed during reproduction. Such functional complexity can indeed be observed in the spermalege. In addition to the wound reduction and sealing functions identified here (see also [17,19]), the spermalege activates sperm [42], digests seminal fluid [42] and reduces sexual infection [18]. If the nature of the female defence strategy is responsible for such a rapid generation of trait diversity and complexity, sexual conflict theory will benefit from a unified approach of considering two female defence strategies, resistance and tolerance, which in other conflict systems has been shown to accelerate diversity even further [20,43].
